# Circulating pentraxin 3 is positively associated with chronic hyperglycemia but negatively associated with plasma aldosterone concentration

**DOI:** 10.1371/journal.pone.0196526

**Published:** 2018-05-01

**Authors:** Yuichi Takashi, Minae Koga, Yoko Matsuzawa, Jun Saito, Masao Omura, Tetsuo Nishikawa

**Affiliations:** Endocrinology and Diabetes Center, Yokohama Rosai Hospital, Yokohama, Kanagawa, Japan; Universita degli Studi di Milano, ITALY

## Abstract

Pentraxin 3 (PTX3) is reported to be a vascular inflammation marker providing prognostic information of vasculopathy. Until today, however, the effect of aldosterone or oxidative stress on the regulation of PTX3 is unknown. In present study, we investigated to find regulative factors, especially aldosterone and oxidative stress, on PTX3. Serum PTX3 levels were measured in 75 patients (45 male and 30 women, aged 55.1±13.4 year-old (mean±SD)) with various endocrine disorders including 47 with diabetes, 24 with primary aldosteronism (PA). All participants were free from cardio vascular diseases and diabetic retinopathy. Serum PTX3 level was significantly lower in patients with PA than without PA and was significantly higher in patients with diabetes than without diabetes. PTX3 was significantly correlated with glycated hemoglobin (HbA1c), urinary albumin excretion (UAE) and plasma aldosterone concentration (PAC) (r = 0.431, P<0.001; r = 0.313, P = 0.009; r = -0.375, P = 0.004). A stepwise multiple regression analysis chose HbA1c and UAE as independent determinants of PTX3 (β = 0.282, P<0.001; β = 0.783, P<0.001). On the other hand, PTX3 was not significantly correlated with HbA1c and UAE but significantly negatively correlated with PAC in patients with diabetes. Therefore, it might be suggested that PTX3 is positively regulated by chronic hyperglycemia but negatively regulated by aldosterone, and is associated with urinary albumin excretion as a micro vasculopathy.

## Introduction

Pentraxins are superfamily of acute-phase proteins that induce short pentraxins such as C-reactive protein (CRP) or long pentraxins such as pentraxin 3 (PTX3) [1]. Unlike CRP, PTX3 is expressed in atherosclerotic lesions, but not in hepatocytes [2–6]. Thus, PTX3 is considered to be a candidate of vascular inflammation marker to evaluate vascular complications. Recently, serum PTX3 level is accepted as a major cardiovascular disease (CVD) risk factor, providing prognostic information of CVD [7–9]. Actually, it was shown that serum PTX3 level was positively correlated with atherosclerotic markers in patients with diabetes [10,11]. Serum PTX3 level was correlated with insulin resistance in patients with obesity or polycystic ovary syndrome [12,13]. PTX3 was also reported to promote insulin sensitivity in obese mice model [14]. In addition, it was reported that PTX3 was a specific marker of ischemic heart disease [15–18] or Takayasu arteritis [19]. On the other hand, PTX3 has atheroprotective effects in several experimental models [20–22]. Furthermore, it was reported that PTX3 was negatively correlated with atherosclerotic markers in patients with obesity [23,24] or gestational diabetes mellitus (GDM) [25]. Therefore, the significance and function of PTX3 as a vascular inflammation marker are still controversial.

Unlike aberrant glucose metabolism, however, little is known about the relationship between PTX3 and other hormones. Various types of endocrine disorder including primary aldosteronism (PA) and Cushing’s syndrome is well known to induce vascular impairment. Especially in primary aldosteronism as an important form of secondary hypertension, aldosterone is associated with increase of CVD. Recently, aldosterone was reported as a mediator of vasculopathy in early-stage of hypertension [26]. It was reported that serum PTX3 level in patients with adrenal adenomas was higher than that of in healthy controls and PTX3 was correlated with urinary metanephrine concentration [27]. On the other hand, they recruited only one patient with PA and didn’t show the relationship between PTX3 and aldosterone. There is no report indicating the association between PTX3 and aldosterone.

We consider that oxidative stress is a common regulative factor on PTX3 in these diseases including diabetes and PA. It was reported that both hyperglycaemia and aldosterone increased oxidative stress [28–30]. In present study, we investigated to find regulative factors, especially aldosterone and oxidative stress, on PTX3.

## Subjects and methods

### Study population

The study protocol was approved by the research ethics committee of Yokohama Rosai Hospital. We recruited a consecutive series of 75 inpatients of Yokohama Rosai Hospital, Yokohama City, Kanagawa, Japan. Each participant provided written informed consent. Forty-seven of them had diabetes including four type 1 and 43 type 2 diabetes. In addition, there were 24 PA, two non-functional adrenal adenomas, a Cushing’s syndrome, a ACTH-independent bilateral adrenocortical macronodular hyperplasia (AIMAH), a prolactinoma, an acromegaly, a renovascular hypertension and a severe osteoporosis. Four patients had both type 2 diabetes and PA. Mean age was 55.1±13.4 years and 45 males and 30 females were included in this study. Hypertension and dyslipidemia were shown in 46 and 24 participants, respectively. All participants are free from prevalence of CVD and diabetic retinopathy.

### Measurement procedures

Fasting blood and early morning second urine samples were collected second day of hospitalization. Serum PTX3 level was measured by LSI Medience Corporation (Tokyo, Japan) with ELISA system [15]. In addition, an oxidative stress marker, urine 8-iso-Prostaglandin F2α (8-epi-PGF2α), was also examined by LSI Medience Corporation (Tokyo, Japan) with ELISA system. Glycated hemoglobin (HbA1c), creatinine, urinary albumin excretion (UAE), urinary sodium excretion, LDL cholesterol, HDL cholesterol, triglyceride, plasm renin activity (PRA), plasma aldosterone concentration (PAC), adrenocorticotropic hormone (ACTH), cortisol and high-sensitivity CRP were measured using standard laboratory protocol. Maximum carotid intima-media thickness (max IMT) and brachial-ankle pulse wave velocity (baPWV) were evaluated using echotomographic system and plethysmography, respectively. The IMT was measured as the distance from the leading edge of the first echogenic line to the leading edge of the second echogenic line [31]. The PWV was calculated as the distance between recording sites measured over the surface of the body, divided by the time interval between the feet of the flow waves [32].

### Statistical analysis

The data are shown as mean±SD. The relationships between PTX3 and various clinical parameters including PAC and 8-epi-PGF2α were examined. Correlations between PTX3 and various atherosclerotic markers and their risk factors were tested by using Spearman’s correlation coefficient because PTX3 was an undistributed variable. A stepwise multiple regression analysis was also used. Non-categorical data were changed into categorical variables. Statistical analysis was performed by BellCurve 2.15 (SSRI, Tokyo, Japan). A P value of <0.05 was considered to be significant.

## Results

The clinical characteristics of participants were shown in Table 1. Systolic and diastolic blood pressure were higher in patients with PA than without PA (P = 0.031, P = 0.019). There was no difference in serum creatinine level between PA and non-PA or diabetes and non-diabetes patients (P = 0.756, P = 0.457). Serum HDL cholesterol level was lower in patients with diabetes than without diabetes (P = 0.001). Moreover, the other lipid profiles did not show any difference between diabetes and non-diabetes patients. Serum ACTH level was higher in patients with non-PA than with PA (P = 0.034), however, there were no significant differences in serum cortisol level between PA and non-PA patients (P = 0.569). Serum high-sensitive CRP level was higher in patients with diabetes (P = 0.003) and max IMT was lower in patients with PA (P = 0.024). Oxidative stress marker, 8-epi-PGF2α was higher in patients without PA or with diabetes than with PA or without diabetes (P = 0.043, P = 0.026). It was reported that 8-epi-PGF2α was a perceptive oxidative stress marker and could reflect not only chronic hyperglycemia but also acute glucose swings in patients with type 2 diabetes [33]. Finally, serum PTX3 level was higher in patients without PA or with diabetes than with PA or without diabetes (P = 0.006, P = 0.003). The use of anti-hypertensive drugs, anti-diabetic drugs and anti-dyslipidemia drugs were also shown in Table 1. Nobody used mineralocorticoid receptor (MR)-blockers nor antiplatelet agents.

**Table 1:** The clinical characteristics of participants.

Parameters	Reference range	Total	PA	non-PA	P value	Diabetes	non-Diabetes	P value
Sex (%male)		60.0	50.0	64.7	0.313	70.2	42.9	0.028*
Age (years)		55.1 ± 13.4	50.8 ± 11.1	57.2 ± 14.0	0.040*	56.8 ± 13.9	52.4 ± 12.3	0.156
Body mass index (kg/m^2^)	18.0–25.0	25.1 ± 4.5	24.7 ± 4.3	25.4 ± 4.6	0.526	25.7 ± 4.9	24.1 ± 3.6	0.109
Existence of hypertension (%)		61.3	100	49.0	<0.001*	51.1	78.6	0.027*
Existence of diabetes (%)		62.7	16.0	76.5	<0.001*	100	0	<0.001*
Existence of dyslipidemia (%)		32.0	16.0	39.2	0.065	36.2	25.0	0.443
Duration of hypertension (years)		5.3 ± 8.5	7.8 ± 8.1	3.9 ± 8.5	0.072	3.7 ± 8.0	7.6 ± 8.7	0.067
Duration of diabetes (years)		5.5 ± 8.2	0.6 ± 1.9	7.7 ± 9.0	<0.001*	8.6 ± 9.0	0.3 ± 1.3	<0.001*
Current smoker (%)		29.6	22.7	32.7	0.575	30.4	28.0	1.000
Systolic blood pressure (mmHg)	<140	128.5 ± 18.7	135.1 ± 15.4	125.5 ± 19.5	0.031*	125.8 ± 20.0	133.3 ± 15.5	0.087
Diastolic blood pressure (mmHg)	<90	77.9 ± 13.4	83.4 ± 12.7	75.3 ± 13.1	0.019*	75.2 ± 13.0	82.7 ± 13.0	0.026*
HbA1c (%)	4.6–6.2	7.7 ± 2.7	5.2 ± 0.7	8.9 ± 2.4	<0.001*	9.1 ± 2.3	5.2 ± 0.5	<0.001*
Creatinine (mg/dl)	0.31–1.10	0.84 ± 0.80	0.81 ± 0.29	0.85 ± 0.95	0.756	0.88 ± 0.99	0.77 ± 0.28	0.457
Urinary albumin excretion (mg/gCre)	<18.0	80.8 ± 320.6	8.0 ± 7.5	117.9 ± 390.3	0.066	120.6 ± 394.4	7.7 ± 7.2	0.064
Urinary sodium excretion (mEq/gCre)		152 ± 200	122 ± 110	172 ± 242	0.297	182 ± 251	117 ± 108	0.198
LDL cholesterol (mg/dl)	70–139	107.6 ± 32.1	107.0 ± 30.9	107.9 ± 32.9	0.914	108.1 ± 32.9	106.6 ± 31.2	0.847
HDL cholesterol (mg/dl)	Male: 40–86 Female: 40–96	50.3 ± 12.8	55.8 ± 13.2	47.7 ± 11.9	0.015*	46.2 ± 10.7	57.3 ± 13.3	0.001*
Triglyceride (mg/dl)	50–149	117.7 ± 61.3	109.3 ± 76.8	121.7 ± 52.8	0.482	124.3 ± 50.9	106.1 ± 75.9	0.272
Plasma renin activity (ng/ml/hour)	0.3–2.9 (supine)	1.31 ± 1.42	0.75 ± 0.85	1.71 ± 1.61	0.005*	1.61 ± 1.71	0.94 ± 0.86	0.057
Plasma aldosterone concentration (pg/ml)	29.9–159 (supine)	105.0 ± 59.4	127.2 ± 75.9	89.4 ± 38.3	0.032*	94.8 ± 42.4	117.5 ± 74.3	0.174
ACTH (pg/ml)	7.2–63.3	28.9 ± 18.5	23.0 ± 13.7	32.6 ± 20.2	0.034*	32.9 ± 18.6	23.7 ± 17.2	0.055
Cortisol (μg/dl)	6.24–18.0	10.5 ± 4.7	10.1 ± 3.1	10.8 ± 5.6	0.569	11.5 ± 5.3	9.4 ± 3.7	0.076
High-sensitivity CRP (mg/dl)	<0.3	0.11 ± 0.12	0.10 ± 0.13	0.11 ± 0.12	0.574	0.14 ± 0.14	0.06 ± 0.06	0.003*
Max IMT (mm)	<1.0	1.65 ± 0.88	1.32 ± 0.78	1.80 ± 0.89	0.024*	1.80 ± 0.88	1.39 ± 0.83	0.052
baPWV (cm/second)		1554 ± 342	1481 ± 280	1594 ± 368	0.177	1603 ± 375	1464 ± 254	0.086
8-epi-PGF2α (pg/mgCre)	107–398	310.7 ±151.5	265.3 ±112.1	332.9 ±164.0	0.043*	337.6 ± 168.5	264.8 ± 104.9	0.026*
PTX3 (ng/ml)	0.57–4.32	2.81 ± 1.71	2.16 ± 1.07	3.11 ± 1.87	0.006*	3.20 ± 1.91	2.15 ± 1.04	0.003*
ARBs (%)		18.7	0	27.5	0.003*	27.7	3.6	0.012*
ACE inhibitors (%)		2.7	0	4.0	1.000	4.3	0	0.526
MR-blockers (%)		0	0	0	-	0	0	-
CCBs (%)		45.3	75.0	31.4	0.001*	36.2	60.7	0.055
Diuretics (%)		5.3	0	7.8	0.299	6.4	3.6	1.000
β-blockers (%)		5.3	0	5.9	0.547	6.4	3.6	1.000
α-blockers (%)		8.0	16.0	3.9	0.079	6.4	10.7	0.665
Number of anti-hypertensive drugs		0.85 ± 1.09	0.95 ± 0.69	0.80 ± 1.23	0.570	0.87 ± 1.26	0.82 ± 0.72	0.846
Insulin (%)		13.3	4.2	17.6	0.154	19.1	0	0.022*
GLP1-RAs (%)		0	0	0	-	0	0	-
DPP4 inhibitors (%)		14.7	4.2	19.6	0.093	23.4	0	0.005*
SGLT2 inhibitors (%)		0	0	0	-	0	0	-
Metformins (%)		28.0	8.3	37.3	0.012*	44.7	0	<0.001*
Thiazolidines (%)		5.3	0	7.8	0.299	8.5	0	0.290
Sulfonylureas (%)		25.3	4.2	35.3	0.004*	40.4	0	<0.001*
Glinides (%)		5.3	0	7.8	0.299	8.5	0	0.290
α-GIs (%)		17.3	0	25.5	0.007*	27.7	0	0.001*
Statins (%)		32.0	16.0	39.2	0.065	36.2	25.0	0.443
Antiplatelet agents (%)		0	0	0	-	0	0	-

PA, primary aldosteronism; HbA1c, glycated hemoglobin; ACTH, adrenocorticotropic hormone; CRP, C-reactive protein; Max IMT, maximum carotid intima-media thickness; baPWV, branchial-ankle pulse wave velocity; 8-epi-PGF2α, 8-iso-Prostaglandin F2α; PTX3, pentraxin 3; ARB, angiotensin receptor blocker; ACE, angiotensin converting enzyme; MR, mineralocorticoid receptor; CCB, calcium channel blocker; GLP1-RAs, glucagon-like peptide-1 receptors agonist; DPP4, dipeptidyl peptidase 4; SGLT2, sodium-glucose cotransporter 2; α-GI, α-glycosidase inhibitors.

* P<0.05

We examined correlations between PTX3 and each parameter in univariate model (Table 2). Serum PTX3 level was significantly positively correlated with high-sensitivity CRP as a non-specific inflammation marker (r = 0.231, P = 0.046). We also found that serum PTX3 level was significantly positively correlated with existence of diabetes, HbA1c and UAE as reported previously in this study (r = 0.379, P<0.001; r = 0.431, P<0.001; r = 0.313, P = 0.009). These significant correlations with PTX3 weren’t shown in patients with PA nor diabetes. PTX3 wasn’t correlated with 8-epi-PGF2α as an oxidative stress marker, however, PTX3 was significantly positively correlated with 8-epi-PGF2α in patients with PA (r = 0.412, P = 0.045). Unexpectedly, PTX3 was significantly negatively correlated with PAC (r = -0.375, P = 0.004). The negative association between PTX3 and PAC was also shown in not PA but diabetic patients (r = -0.004, P = 0.986; r = -0.450, P = 0.010). On the other hand, PTX3 wasn’t correlated with urinary sodium excretion. PTX3 wasn’t shown to correlate with cortisol as another steroid hormone. In addition, PTX3 wasn’t associated with BMI, existence of hypertension or dyslipidemia, duration of hypertension or diabetes, smoking, serum creatinine level, max IMT, baPWV and the use of each drug or the number of anti-hypertensive drugs.

**Table 2:** Correlations between PTX3 and each parameter.

	Total		PA		Diabetes	
	r	P	r	P	r	P
Sex (male/female)	-0.006	0.962	-0.060	0.780	0.185	0.213
Age (years)	-0.025	0.831	-0.248	0.242	-0.064	0.672
Body mass index (kg/m^2^)	-0.050	0.671	-0.191	0.370	-0.026	0.861
Existence of hypertension	-0.135	0.249	-	-	-0.159	0.287
Existence of diabetes	0.379	<0.001*	-0.210	0.325	-	-
Existence of dyslipidemia	-0.086	0.464	0.162	0.451	-0.171	0.249
Duration of hypertension (years)	-0.172	0.160	0.114	0.595	-0.206	0.196
Duration of diabetes (years)	0.216	0.066	0.092	0.676	-0.211	0.159
Current smoker	0.011	0.926	0.222	0.320	0.041	0.787
Systolic blood pressure (mmHg)	0.052	0.669	0.257	0.248	0.096	0.537
Diastolic blood pressure (mmHg)	-0.008	0.950	0.321	0.146	-0.022	0.890
HbA1c (%)	0.431	<0.001*	-0.239	0.272	0.290	0.051
Creatinine (mg/dl)	-0.133	0.255	-0.085	0.693	-0.112	0.452
Urinary albumin excretion (mg/gCre)	0.313	0.009*	0.074	0.737	0.182	0.237
Urinary sodium excretion (mEq/gCre)	0.051	0.705	0.058	0.793	-0.260	0.158
LDL-cholesterol (mg/dl)	-0.094	0.430	-0.309	0.142	-0.061	0.687
HDL-cholesterol (mg/dl)	-0.075	0.524	0.328	0.117	0.069	0.646
Triglyceride (mg/dl)	-0.191	0.103	-0.418	0.042*	-0.294	0.045*
Plasma renin activity (ng/ml/hour)	0.005	0.971	-0.105	0.627	-0.041	0.824
Plasma aldosterone concentration (pg/ml)	-0.375	0.004*	-0.004	0.986	-0.450	0.010*
ACTH (pg/ml)	0.220	0.095	-0.012	0.957	0.211	0.239
Cortisol (μg/dl)	0.155	0.238	0.031	0.888	0.183	0.307
High-sensitivity CRP (mg/dl)	0.231	0.046*	0.281	0.184	0.114	0.447
Max IMT (mm)	0.229	0.053	0.375	0.078	0.098	0.519
baPWV (cm/second)	-0.046	0.724	-0.391	0.072	-0.168	0.295
8-epi-PGF2α (pg/mgCre)	0.145	0.223	0.412	0.045*	0.101	0.506
ARBs	0.074	0.526	-	-	-0.025	0.870
ACE inhibitors	0.119	0.311	-	-	0.109	0.467
CCBs	-0.205	0.078	-0.083	0.698	-0.282	0.055
Diuretics	0.003	0.981	-	-	0.051	0.732
β-blockers	0.181	0.120	0.136	0.528	0.225	0.129
α-blockers	-0.009	0.938	0.024	0.911	0.141	0.343
Number of anti-hypertensive drugs	-0.109	0.354	-0.003	0.989	-0.117	0.433
Insulin	0.178	0.128	-	-	0.104	0.488
DPP4 inhibitors	0.134	0.252	-	-	<0.001	1.000
Metformins	0.087	0.457	-	-	-0.175	0.239
Thiazolidines	0.038	0.744	-	-	-0.039	0.793
Sulfonylureas	0.154	0.186	-	-	-0.011	0.941
Glinides	0.212	0.067	-	-	0.205	0.167
α-GIs	0.020	0.868	-	-	-0.202	0.174
Statins	-0.086	0.464	0.162	0.451	-0.171	0.249

Sex: male = 1, female = 0; Existence of each disease or use of each drug: yes = 1, no = 0; PTX3, pentraxin 3; PA, primary aldosteronism; HbA1c, glycated hemoglobin; ACTH, adrenocorticotropic hormone; CRP, C-reactive protein; Max IMT, maximum carotid intima-media thickness; baPWV, branchial-ankle pulse wave velocity; 8-epi-PGF2α, 8-iso-Prostaglandin F2α; ARB, angiotensin receptor blocker; ACE, angiotensin converting enzyme; CCB, calcium channel blocker; DPP4, dipeptidyl peptidase 4; α-GI, α-glycosidase inhibitors.

* P<0.05

Furthermore, a stepwise multiple regression analysis was performed between PTX3 and each parameter which was correlated with serum PTX3 level in univariate model, such as existence of diabetes, HbA1c, UAE, PAC, high-sensitivity CRP. This analysis chose HbA1c and UAE as independent determinants of PTX3 (β = 0.282, P<0.001; β = 0.783, P<0.001) (Table 3).

**Table 3:** Stepwise multiple regression analysis between PTX3 and each parameter.

	β	P
HbA1c (%)	0.282	<0.001
Urinary albumin excretion (mg/gCre)	0.738	<0.001

*R*^*2*^ = 0.69

Existence of diabetes, HbA1c, urinary albumin excretion, plasma aldosterone concentration, high-sensitivity CRP were used in the stepwise multiple regression analysis.

β: standard partial regression coefficient; PTX3, pentraxin 3; HbA1c, glycated hemoglobin; CRP, C-reactive protein.

## Discussion

We have shown the significant correlation between PTX3 and existence of diabetes, HbA1c, UAE, PAC and high-sensitive CRP in total population of this study. In addition, PTX3 was significantly correlated with triglyceride and 8-epi-PGF2α in patients with PA, and with triglyceride and PAC in patients with diabetes. First, in this study, PTX3 was positively correlated with existence of diabetes and HbA1c. Chronic hyperglycemia seems to increase serum PTX3 level. Moreover, PTX3 was positively correlated with UAE. Microalbuminuria was known to one of the earliest clinical detectable marker for vascular impairment [34]. Although we could find the significant correlation between PTX3 and high-sensitivity CRP, high-sensitivity CRP wasn’t shown to correlate with UAE. Therefore, it is suggested that PTX3 is more closely associated with at least micro-vascular damage. However, PTX3 wasn’t correlated with max IMT nor baPWV as a macro-vascular impairment marker. Therefore, we could consider that PTX3 is insufficient as a vascular inflammation marker. Another report showed that PTX3 concentration was correlated with mean IMT [27]. Because healthy controls were enrolled in this study, they could observe the relationship between PTX3 and mean IMT. Our study did not include healthy controls. We considered that we could not evaluate the progression of macro-vascular damage among patients with PA or diabetes by using PTX3. On the other hand, negative correlations between serum PTX3 level and atherosclerotic markers were also reported [23–25,35]. For example, serum PTX3 level was negatively correlated with baPWV in patients with obesity [23,24] and GDM [25]. In addition, cardioprotective function of PTX3 was shown in several experimental models both *in vivo* and *vitro* [20–22]. In passing, PTX3 deficient mice develop larger atherosclerosis [21]. The precise role of PTX3 in vascular diseases was indeed unknown.

Then, our study newly revealed that PTX3 was negatively correlated with PAC and the correlation was also shown in patients with diabetes. Although we could not observe the relationship between PTX3 and PAC in patients with PA, it might be considered that abnormal regulation of PAC in PA canceled the relationship with PTX3. There was no significant difference in serum PTX3 level between PA and non-PA patients without diabetes to remove the influence of chronic hyperglycemia (2.22±1.12 vs 1.99±0.86 ng/ml, P = 0.607). However, it is difficult to evaluate this result because there were only eight patients without PA and diabetes. There is a possibility to exist some interactions between PAC and chronic hyperglycemia. It was well known that excess action of aldosterone induced vascular complications. Recently, aldosterone was reported as a mediator of vasculopathy in early-stage of hypertension [26]. Furthermore, it was reported that PTX3 was increased in patients with adrenal adenoma [27]. The positive correlation between PTX3 and urinary metanephrine level was also shown. Although we didn’t evaluate the state of catecholamine, we expected at least positive correlation between PTX3 and PAC in current study. As far as we know, there is no previous report indicating the relationship between PTX3 and PAC. Thus, it is just speculation for this phenomenon that aldosterone decreases PTX3 production in blood vessels and inhibits cardioprotective effect via PTX3. This is one possible mechanism of aldosterone-induced vasculopathy characterized by necrosis and fibrosis. Aldosterone might be a novel negative regulator of PTX3 at least in this model. This effect wasn’t shown in cortisol as another steroid hormone secreted by adrenal glands, however, we didn’t evaluate the overproduction of cortisol by dexamethasone suppression test in present study.

Furthermore, PTX3 was also positively correlated with oxidative stress marker, 8-epi-PGF2α in patients with PA. It was reported that 8-epi-PGF2α was a perceptive oxidative stress marker among all of oxidative stress marker, for example, 8-epi-PGF2α could reflect not only chronic hyperglycemia but also acute glucose swings in patients with type 2 diabetes [33]. The toxic effect of reactive oxygen species (ROS) lead to damage to blood vessels [36,37] and 8-epi- PGF2α is a marker of oxidative damage to DNA. Hyperglycemia and aldosterone increase oxidative stress [28–30]. Because the control of blood glucose was poor in patients with diabetes whose mean HbA1c was 9.1±2.3%, we could not observe the relationship between PTX3 and 8-epi-PGF2α in total population or patients with diabetes. It is suggested that PTX3 also reflects the injuring blood vessels by ROS.

In present study, patients with various endocrine disorders other than PA and diabetes were included. Although serum PTX3 levels in patients with non-functional adrenal adenoma, acromegaly, renovascular hypertension and severe osteoporosis were under the first quartile, we could not observe certain trend in other diseases.

In conclusion, serum PTX3 level reflected chronic hyperglycemia and microalbuminuria. Aldosterone was uncovered to be a novel negative regulator of PTX3. However, the present study had several limitations. First, we included patients with various endocrine disorders as a consecutive series of patients. Therefore, the study had a substantial selection bias in inclusion criteria. Second, this study was cross-sectional small number study without healthy control subjects. Further large number study is needed. Third, we didn’t exclude the influence of every medication such as anti-diabetes, hypertension, dyslipidemia agents. It was reported that these medications could modify serum PTX3 level [38–41]. Another prospective study should be performed.

## Supporting information

S1 File Minimal data set of this study. (XLSX)
